# Investigation of feeding and nutritional problems related to long-term enteral nutrition support among children with disabilities: a pilot study

**DOI:** 10.3389/fnut.2025.1672436

**Published:** 2025-11-18

**Authors:** Sara Zaher, Hadeel Aldhowayan, Asmaa Almalki, Riman Alsaedi, Ruba Almghamsi, Lujain Aljazaeri, Walaa Abdullah Mumena

**Affiliations:** Clinical Nutrition Department, College of Applied Medical Sciences, Taibah University, Madinah, Saudi Arabia

**Keywords:** paediatrics, disabled children, feeding problems, enteral nutrition, nutrition support

## Abstract

**Background:**

Enteral Nutrition (EN) is considered a standard intervention for patients with disabilities who cannot meet their nutritional requirements orally and are at risk for malnutrition secondary to eating difficulties. The current study examined common feeding and nutritional problems related to prolonged EN among disabled children.

**Methods:**

A cross-sectional, pilot study was conducted in Saudi Arabia between December 2023 and March 2024. Caregivers of children with disabilities were invited to complete an online questionnaire that gathered demographic data and explored feeding difficulties and challenges related to enteral nutrition.

**Results:**

A total of 41 caregivers completed the survey regarding their children. The median age (IQR) of disabled children was 3.2 (1.7–6.6) years. The most frequently reported feeding and nutritional problems in this cohort were constipation [median = 3.0, IQR: 2.0–4.0], weight loss [median = 3.0, IQR: 1.0–4.0], and gastroesophageal reflux [median = 2.0, IQR: 1.0–3.0].The regression analysis showed a statistical association between the indication for nutrition support and the subsequent detected feeding/nutritional problem, *p-value*<*0.05*. It also showed that the primary diagnosis (*r* = 0.459, *p-value* = 0.003) and health status (*r* = 0.458, *p-value* = 0.003) were statistically significant predictors of the frequency of reported feeding and nutritional problems among this children group. Additionally, the challenges experienced by the caregivers were statistically related to the type of EN provided (*r* = 0.491, *p-value* = *0.001*).

**Conclusion:**

The study provided insight into the typical feeding and nutritional problems associated with long-term EN among children with disabilities. Identifying these issues can support early diagnosis and the implementation of appropriate nutritional interventions, ultimately helping to optimize growth and improve quality of life for these children.

## 1 Introduction

Pediatric feeding disorders are complex conditions that impair a child's ability to consume adequate nutrition necessary for optimal growth and development. They encompass difficulties related to eating and swallowing that may stem from medical condition (1, 2). Children with developmental disabilities and neurological impairments are particularly vulnerable to feeding problems, as these conditions often compromise oral–motor coordination, swallowing safety, and gastrointestinal function (3).

Disability remains a major global health concern, affecting more than 15% of the world's population (4). In Saudi Arabia, the prevalence of disability is 3,326 per 100,000 people, accounting for approximately 3.3% of the total population. Among those affected, 23% are children and adolescents under the age of 15 (4). Pediatric disabilities such as cerebral palsy (CP), craniofacial anomalies (e.g., cleft lip or palate), and other conditions place children at particularly high risk of malnutrition, often due to oral–motor feeding and swallowing difficulties (3). Swallowing difficulties, chewing problems, and gastrointestinal issues are common challenges among the pediatric disabled population. These issues hinder adequate oral intake and increase the risk of malnutrition secondary to feeding difficulties (5–7). Chronic insufficient oral intake can lead to growth retardation, failure to thrive, and compromised immune function, all of which negatively affect the wellbeing and quality of life of children with disabilities (3, 8, 9). Therefore, the early implementation of diagnostic tools and timely nutritional interventions is essential to address feeding problems in this vulnerable population.

Enteral nutrition (EN) is widely accepted as a standard method of nutritional support for children with disabilities who are unable to meet at least 60% of their nutritional needs through oral intake (9–11). The European Society for Pediatric Gastroenterology, Hepatology, and Nutrition (ESPGHAN) defines EN as the delivery of nutrients either in the form of a liquid formula or a blenderized diet to the stomach, duodenum, or jejunum via a feeding tube or an artificial opening in the abdomen (5). Indications for initiating EN in children with disabilities include inadequate oral intake, developmental delays, gastrointestinal dysfunction, increased nutritional requirements, and nutrient losses secondary to vomiting (5). Undeniably, enteral feeding is considered a life-saving intervention for this vulnerable population. It offers numerous benefits, such as promoting optimal growth, preventing malnutrition, maintaining adequate hydration, supporting special dietary needs (e.g., in children with metabolic disorders), and reducing the risk of aspiration associated with gastroesophageal reflux disease (GERD), which is common among children with disabilities (9).

It has been reported that a considerable number of disabled children rely on long-term EN, over 6 months, to maintain their nutritional requirements (12, 13). When EN is properly implemented, it can significantly enhance the health-related quality of life for these children (3). However, prolonged EN is often associated with various tube-feeding-related complications that may pose serious health risks, regardless of the feeding route used (14–16). Therefore, close monitoring and assessments is required by both the caregivers and healthcare professionals to ensure early detection of any EN related complications (7, 17). The most common problems associated with long-term EN include infection at the insertion site, vomiting, and diarrhea which they need immediate intervention and careful home care (14). Understanding these challenges is crucial for caregivers and healthcare providers to ensure that children with disabilities receive optimal nutritional support and care, helping to sustain growth and improve their overall quality of life.

Currently, there is a lack of comprehensive studies exploring the overall experience of prolonged tube feeding among children with disabilities from the caregivers' perspective (15). This gap is especially evident in the context of Saudi Arabia, where related challenges and nutritional problems remain underexamined. Therefore, this study was set out to investigate feeding and nutritional issues that are related to long-term EN among disabled children in Saudi Arabia.

## 2 Materials and methods

### 2.1 Study design and sampling

**Figure 1:**
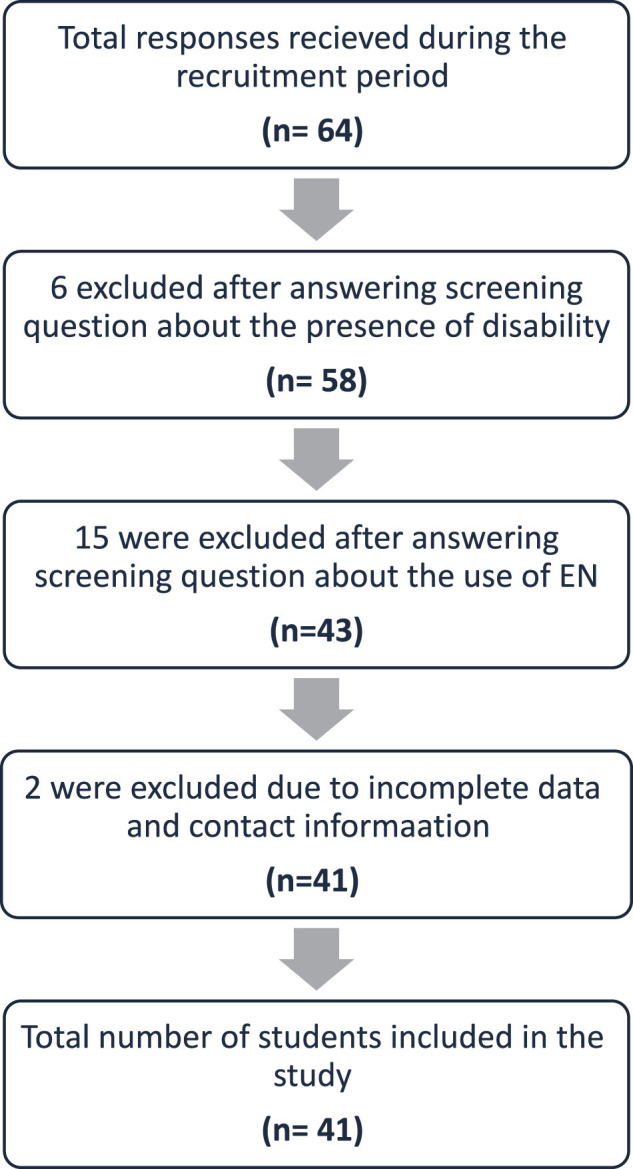
Flowchart of study sample.

In this cross-sectional pilot study, all caregivers of children with disabilities aged 1 to 18 years who were receiving long-term enteral nutrition (EN) and residing in Saudi Arabia were eligible to participate. Given the exploratory nature of this study, no formal sample size calculation was performed. Accordingly, participant recruitment was guided by feasibility and accessibility within the study setting (Figure 1).

The study aimed to gather information on nutritional issues and feeding challenges associated with long-term EN in this population. Convenience sampling was initially used, as data were collected through an online survey distributed via various social media platforms, including WhatsApp and X (formerly Twitter), between December 2023 and March 2024. Chain referral sampling was subsequently employed to enhance participant recruitment and ensure an adequate sample size.

The study received ethical approval from the Ethics Committee at Taibah University (Certificate No. 2024/177/203 CLN). An information sheet outlining the study's purpose, procedures, and confidentiality assurances was provided on the first page of the online survey. Informed consent was obtained electronically through a mandatory question requiring participants to confirm their agreement to participate before proceeding with the questionnaire.

### 2.2 Questionnaire development and validation

A detailed description of the questionnaire development process is provided in in a study conducted by Zaher and Ajabnoor (18). In brief, the questionnaire included 15 questions related to sociodemographic information of the children and caregivers and 15 questions related to the nutritional status and other health-related conditions of the children. The last section of the questionnaire included 10 items to assess the feeding and nutritional problems associated with EN among disabled children and an additional two questions to evaluate the level of feeding difficulties experienced by the caregivers. The participants were asked to rate the frequency of each item on a scale from 1 (Never), 2 (Rarely), 3 (Sometimes), 4 (Often) to 5 (Always) (18).

### 2.3 Statistical analysis

Data were analyzed using the Statistical Package for the Social Sciences (SPSS) software, version 22 (SPSS Inc., Chicago, IL, USA). The normality of continuous variables was assessed using the Shapiro–Wilk test. Descriptive statistics were reported as frequencies and percentages for categorical variables, while continuous variables were presented as mean ± standard deviation (SD) and median with interquartile range (IQR), as appropriate. For each participant, a total Likert scale score was calculated for feeding and nutritional problems, as well as a separate total score for caregiver-reported challenges, to facilitate statistical analysis.

Linear regression analysis was conducted to identify factors influencing feeding frequency and nutritional problems among children with disabilities. Additionally, regression analysis was used to examine factors associated with the challenges faced by caregivers in providing and managing EN. A *p-value* of < 0.05 was considered statistically significant.

## 3 Results

Forty-one children receiving long-term EN were included in this study. The median age of the children was 3.2 years (IQR: 1.7–6.6), with a slightly higher proportion of females (53.6%) compared to males (46.3%). The most common diagnosis was CP (43.9%), followed by mental disabilities (19.2%), learning difficulties (12.2%), attention deficit hyperactivity disorder (7.3%), and autism spectrum disorder (4.9%). These children were from various regions across Saudi Arabia, with the majority residing in the central region (43.2%) (Table 1).

**Table 1:** General characteristics of the study participants.

**Caregivers (*****n*** = **41)**		
The primary caregiver	Mother	40 (97.5%)
	Father	0 (0.05%)
	Brother / sister	0
	Grandfather / grandmother	0
	Another family member	0
	Nurse	1
Age (Years)	Median (IQR)	33 (30–40)
Region	Central region	19 (43.2%)
	Eastern region	10 (24.3%)
	Western region	7 (17.0%)
	Southern region	4 (9.7%)
	Northern region	1 (2.4%)
Educational level	Less than a high school or diploma	8 (19.5%)
	High school or diploma	13 (31.7%)
	Bachelor's degree	19 (46.3%)
	Postgraduate	1 (2.4%)
**Children (*****n*** = **41)**		
Gender	Male	19 (46.3%)
	Female	22 (53.6%)
Age (Years)	Median (IQR)	3.2 (1.7–6.6)
Average sleeping hours	Median (IQR)	9 (6–11)
Family income (Saudi Riyal)	Less than 4,000	11 (26.2%)
	4,000-6,000	9 (21.9%)
	6,000-10,000	7 (17.0%)
	10,000-15,000	10 (24.3%)
	more than 15,000	4 (9.7%)
Order of child in family	Only child in the family	10 (24.3%)
	Oldest child	4 (9.7%)
	Middle child	9 (21.9%)
	The youngest child	18 (43.9%)
Living situation	Lives with both parents	40 (97.5%)
	Lives with mother	1 (0.5%)
	Lives with father	0
	Lives with another family member	0
	Lives in a rehabilitation centre	0
Type of disability^*^	Cerebral Palsy	18 (34.62%)
	Mental Disabilities	10 (19.23%)
	Learning Difficulties	5 (9.62%)
	Attention Deficit Hyperactivity Disorder	3 (5.77%)
	Autism Spectrum Disorder	2 (3.85%)
	Other	14 (26.92%)
Dental problems	Yes	12 (29.2%)
	No	29 (70.7%)
Average number of dietitian visits	Does not visit a dietitian	15 (36.5%)
	Once a year	1 (2.4%)
	Once every 6 months	8 (19.5%)
	Once every 3 months	8 (19.5%)
	Once a month	9 (21.9%)

Values presented as frequencies and percentages.

^*^Participants were allowed to choose more than one diagnosis for children with multiple disabilities.

Caregivers reported various feeding and nutritional problems associated with EN, with the most frequently reported issues being constipation [median 3.0 (IQR: 2.0–4.0)], weight loss [3.0 (1.0–4.0)], and gastroesophageal reflux [2.0 (1.0–3.0)]. Tube-related complications were less commonly reported among the study cohort. Approximately 61% of participants indicated that their children had never experienced tube occlusion or obstruction, and 48.8% reported no instances of insertion site infections (Table 2). The results also highlighted challenges faced by caregivers in determining their children's nutritional needs. About 25% of participants reported frequent (often/always) difficulty in assessing their children's dietary requirements [1.0 (1.0–2.0)]. Regarding tube feeding maintenance, 14.6% of caregivers indicated that they sometimes struggled with tasks such as handling feeding accessories and cleaning the stoma site [3.0 (1.0–3.5)] (Table 2).

**Table 2:** Description of the questionnaire items assessing the frequency of feeding/nutritional problems and feeding challenges associated with long-term EN among children with disabilities.

**Item**		**Likert rating**					**Mean (±SD) Likert rating**	**Median (IQR) Likert rating**
		**1 (Never)** ***n*** **(%)**	**2 (Rarely)** ***n*** **(%)**	**3 (Sometimes)** ***n*** **(%)**	**4 (Often)** ***n*** **(%)**	**5 (Always)** ***n*** **(%)**		
**Nutritional and feeding problems**								
**Tube related problems**	1. How often does your child experience tube leakage from the stoma site?	16 (39.0%)	14 (34.1%)	1126.8%)	0	0	1.88 (**±**0.81)	2.0 (1.0–3.0)
	2. How often does your child experience tube occlusion or obstruction?	25 (61.0%)	8 (19.5%)	8 (19.5%)	0	0	1.59 (**±**0.80)	1.0 (1.0–2.0)
	3. How often does your child experience infection of the tube insertion site?	20 (48.8%)	10 (24.4%)	7 (17.1%)	1 (2.4%)	3 (7.3%)	1.95 (**±**1.20)	2.0 (1.0–3.0)
**Gastrointestinal related problems**	4. How often does your child experience Dumping syndrome (Group of symptoms including diarrhea and sweating caused by rapid gastric emptying) after the delivery of feeding?	31 (75.6%)	4 (9.8%)	5 (12.2%)	1 (2.4%)	0	1.41 (**±**0.80)	1.0 (1.0–1.50)
	5. How often does your child experience vomiting after the delivery of enteral feeding?	15 (36.6%)	7 (17.1%)	15 (36.6%)	2 (4.9%)	2 (4.9%)	2.24 (**±**1.15)	2.0 (1.0–2.0)
	6. How often does your child experience Gastroesophageal reflux (A condition in which the stomach contents leak backward from the stomach into the oesophagus) after the delivery of enteral feeding?	14 (34.1%)	9 (22.0%)	19 (24.4%)	5 (12.2%)	3 (7.3%)	2.37 (**±**1.28)	2.0 (1.0–3.0)
	7. How often does your child experience diarrhea (The passage of 3 or more loose or liquid stools per day (or more frequent passage than is normal)?	18 (43.9%)	9 (22.0%)	8 (19.5%)	4 (9.8%)	2 (4.9%)	2.10 (**±**1.22)	2.0 (1.0–3.0)
	8. How often does your child experience Constipation (Infrequent defecation, painful defecation, or both)?	8 (19.5%)	8 (19.5%)	14 (34.1%)	4 (9.8%)	7 (17.1%)	2.85 (**±**1.33)	3.0 (2.0–4.0)
**Nutritional status related problems**	9. How often does your child experience weight loss?	13 (31.7%)	6 (14.6%)	11 (26.8%)	5 (12.2%)	6 (14.6%)	2.63 (**±**1.42)	3.0 (1.0–4.0)
	10. How often does your child experience weight gain?	30 (73.2%)	5 (12.2%)	3 (7.3%)	2 (4.9%)	1 (2.4%)	1.51 (**±**1.0)	1.0 (1.0–2.0)
**Feeding challenges experienced by caregivers**								
	11. Do you find it difficult to determine your child's nutritional requirements? (Example: Calories and protein)	13 (31.7%)	8 (19.5%)	10 (24.4%)	5 (12.2%)	5 (12.2%)	2.54 (**±**1.38)	2.0 (1.0–3.5)
	12. Do you find it difficult to provide and handle tube feeding for your child? (Example: How to connect and disconnect the feeding tube accessory, clean stoma site...)	28 (68.3%)	4 (9.8%)	6 (14.6%)	3 (7.3%)	0	1.61 (**±**0.997)	1.0 (1.0–2.0)

Values presented as frequencies and percentages.

Regression analysis revealed a statistically significant correlation between the indication for nutrition support and the category of feeding/nutritional problems. Children who were prescribed EN due to inadequate oral intake experienced more tube-related complications (*p-value* < 0.027) (Table 3). Further analysis examining the association between EN indications and specific types of feeding/nutritional problems showed that inadequate oral intake was a significant predictor of insertion site infection, while GERD significantly predicted vomiting following enteral feeding (Table 4). Additionally, both the primary diagnosis (*r* = 0.459, *p-value* = 0.003) and overall health status (*r* = 0.458, *p-value* = 0.003) were significant predictors of the frequency of reported feeding and nutritional problems among this group of children. Specifically, children diagnosed with CP experienced a higher frequency of such problems (*r* = 0.459, *p-value* = 0.003), and dental issues were also significantly associated with increased feeding difficulties (*r* = 0.458, *p-value* = 0.003) (Table 5). Moreover, the challenges faced by caregivers were significantly associated with the type of EN provided (*r* = 0.491, *p-value* = 0.001; Table 6).

**Table 3:** Linear regression analysis to investigate the association between the indication for nutrition support and the frequency of feeding/nutritional problems categories.

**Model 1 (All feeding/nutritional problems)**	***R***	***R^2^***	**Adjusted** ***R**^**2**^*	
**Outcome variable:** Total Likert rating score for all feeding/nutritional problems	**–**	**–**	**–**	
***Dependent variables (n** **=** **41)***	***Beta***	***Partial correlation***	***95%CI***	***P-value***
Not eating enough food orally^b^	–	0.023	–	0.444
Dysphagia ^b^	–	0.092	–	0.283
Gastroesophageal reflux disease ^b^	–	−0.061	–	0.352
Constant choking while eating^b^	–	0.068	–	0.337
**Model 2 (Tube related nutritional problems)**	***R***	***R** ^2^*	**Adjusted** ***R**^2^*	
**Outcome variable:** Total Likert rating score for tube related nutritional problems	–	–	–	
***Dependent variables (n** **=** **41)***	***Beta***	***Partial correlation***	***95%CI***	***P-value***
Not eating enough food orally^b^	–	−0.304	–	0.027
Dysphagia ^b^	–	0.166	–	0.150
Gastroesophageal reflux disease ^b^	–	−0.028	–	0.432
Constant choking while eating ^b^	–	0.117	–	0.233
**Model 3 (Gastro-intestinal nutritional problem)**	***R***	***R** ^2^*	**Adjusted** ***R**^2^*	
**Outcome variable:** Total Likert rating score for gastro-intestinal nutritional problem	**–**	**–**	**–**	
***Dependent variables (n** **=** **41)***	***Beta***	***Partial correlation***	***95%CI***	***P-value***
Not eating enough food orally^b^	–	0.130	–	0.210
Dysphagia^b^	–	0.015	–	0.462
Gastroesophageal reflux disease^b^	–	−0.106	–	0.256
Constant choking while eating^b^	–	0.041	–	0.400
**Model 4 (Nutritional status related problems)**	***R***	***R** ^2^*	**Adjusted** ***R**^2^*	
**Outcome variable:** Total Likert rating score for nutritional status related problems	–	–	–	
***Dependent variables (n** **=** **41)***	***Beta***	***Partial correlation***	***95%CI***	***P-value***
Not eating enough food orally^b^	–	0.199	–	0.106
Dysphagia^b^	–	0.048	–	0.382
Gastroesophageal reflux disease^b^	–	0.063	–	0.347
Constant choking while eating^b^	–	−0.022	–	0.445

^a^Predictors: (constant).

^b^Excluded variables.

All models are adjusted for child's age and gender.

^*^P-value is statistically significant at < 0.05 level.

**Table 4:** Linear regression analysis to investigate the association between the indication of enteral nutrition and the subsequent reported type of feeding/ nutritional problem.

**Model 1 (Infection of the tube insertion site)**	***R***	***R^2^***	**Adjusted** ***R**^**2**^*	
**Outcome variable:** Likert rating score of the Infection of the tube insertion site	0.329	0.109	0.086	
***Dependent variables (n** **=** **41)***	***Beta***	***Partial correlation***	***95%CI***	***P-value***
Not eating enough food orally^a^	−0.734	−0.327	−1.5 to −0.056	0.035^*^
Dysphagia^b^	0.040	0.042	–	0.799
Gastroesophageal reflux disease^b^	−0.042	−0.044	–	0.788
Constant choking while eating^b^	0.115	0.119	–	0.463
**Model 2 (Vomiting after the delivery of enteral feeding)**	***R***	***R** ^2^*	**Adjusted** ***R**^2^*	
**Outcome variable:** Likert rating score of vomiting after the delivery of enteral feeding	0.332	0.110	0.87	
***Dependent variables (n** **=** **41)***	***Beta***	***Partial correlation***	***95%CI***	***P-value***
Gastroesophageal reflux disease^a^	−0.833	−0.332	−1.6 to −0.066	0.034^*^
Not eating enough food orally^b^	−0.007	−0.008	–	0.963
Dysphagia^b^	−0.071	−0.066	–	0.687
Constant choking while eating^b^	0.014	0.011	–	0.948

^a^Predictors: (constant).

^b^Excluded variables.

All models are adjusted for child's age and gender.

^*^P-value is statistically significant at < 0.05 level.

**Table 5:** Linear regression analysis to investigate factors affecting the frequency of feeding and nutritional problems.

**Model 1 (Demographics of the caregivers)**	***R***	***R^2^***	**Adjusted** ***R**^**2**^*	
**Outcome variable:** Total Likert rating score for all feeding/nutritional problems	**–**	**–**	**–**	
***Dependent variables (n** **=** **41)***	***Beta***	***Partial correlation***	***95%CI***	***P-value***
Age of the caregiver^b^	–	−0.305	–	0.026
Educational level^b^	–	−0.118	–	0.232
Financial satiation of the family^b^	–	−0.063	–	0.347
**Model 2 (Diagnosis)**	***R***	***R** ^2^*	**Adjusted** ***R**^2^*	
**Outcome variable:** Total Likert rating score for all feeding/nutritional problems	0.459	0.210	0.190	
***Dependent variables (n** **=** **41)***	***Beta***	***Partial correlation***	***95%CI***	***P-value***
Cerebral palsy^a^	4.490	0.459	1.6 to 7.3	0.003^*^
Mental disabilities^b^	0.154	0.153	–	0.345
Learning difficulties^b^	−0.066	−0.07	–	0.668
Attention Deficit Hyperactivity disorder^b^	−0.096	−0.105	–	0.518
Autism spectrum disorder^b^	0.142	0.157	–	0.333
**Model 3 (Childs health status)**	***R***	***R** ^2^*	**Adjusted** ***R**^2^*	
**Outcome variable:** Total Likert rating score for all feeding/nutritional problems	0.458	0.210	0.190	
***Dependent variables (n** **=** **41)***	***Beta***	***Partial correlation***	***95%CI***	***P-value***
Presence of dental problems^a^	0.489	0.458	1.8 to 7.7	0.003^*^
Presence of food allergy^b^	0.194	0.215	–	0.184
Sleeping hours^b^	−0.050	−0.056	–	0.732
**Model 4 (Child's nutritional care)**	***R***	***R** ^2^*	**Adjusted** ***R**^2^*	
**Outcome variable:** Total Likert rating score for all feeding/nutritional problems	**–**	**–**	**–**	
***Dependent variables (n** **=** **41)***	***Beta***	***Partial correlation***	***95%CI***	***P-value***
Frequency of dietitian's visits^b^	–	0.150	–	0.175
Supplement intake^b^	–	0.153	–	0.169
Type of enteral feeding received (blended food or formula)^b^	–	0.186	–	0.122

^a^Predictors: (constant).

^b^Excluded variables.

All models are adjusted for child's age and gender.

^*^P-value is statistically significant at < 0.05 level.

**Table 6:** Regression analysis to investigate factors affecting the challenges experienced by the caregivers when providing and handling enteral nutrition.

**Model 1 (Demographics of the caregivers)**	***R***	***R^2^***	**Adjusted** ***R**^**2**^*	
**Outcome variable:** Total Likert rating score of caregiver-reported challenges	**–**	**–**	**–**	
***Dependent variable (n** **=** **41)***	***Beta***	***Partial correlation***	***95%CI***	***P-value***
Age of the caregiver^b^	–	−0.191	–	0.116
Educational Level^b^	–	0.077	–	0.315
Financial satiation of the family^b^	–	−0.119	–	0.229
**Model 2 (Diagnosis)**	***R***	***R** ^2^*	**Adjusted** ***R**^2^*	
**Outcome variable:** Total Likert rating score of caregiver-reported challenges	–	–	–	
***Dependent variable (n** **=** **41)***	***Beta***	***Partial correlation***	***95%CI***	***P-value***
Cerebral palsy^b^	–	−0.041	–	0.401
Mental disabilities^b^	–	0.020	–	0.450
Learning difficulties^b^	–	0.048	–	0.348
Attention Deficit Hyperactivity disorder^b^	–	0.121	–	0.226
Autism spectrum disorder^b^	–	−0.074	–	0.323
**Model 3 (Childs health status)**	***R***	***R** ^2^*	**Adjusted** ***R**^2^*	
**Outcome variable:** Total Likert rating score of caregiver-reported challenges	**–**	**–**	**–**	
***Dependent variable (n** **=** **41)***	***Beta***	***Partial correlation***	***95%CI***	***P-value***
Presence of food allergy^b^	–	0.034	–	0.417
Presence of dental problems^b^	–	0.109	–	0.248
Sleeping hours^b^	–	0.134	–	0.201
**Model 4 (Child's nutritional care)**	***R***	***R** ^2^*	**Adjusted** ***R**^2^*	
**Outcome variable:** Total Likert rating score of caregiver-reported challenges	0.491	0.491	0.222	
***Dependent variable (n** **=** **41)***	***Beta***	***Partial correlation***	***95%CI***	***P-value***
Type of enteral feeding received (blended food vs. formula)^a^	2.45	0.492	1.4 to 3.8	0.001^*^
Frequency of dietitian's visits^b^	−0.212	−0.242	–	0.132
Supplement intake^b^	0.070	0.080	–	0.623
**Model 4 (Type of enteral Feeding received)**	***R***	***R** ^2^*	**Adjusted** ***R**^2^*	
**Outcome variable:** Total Likert rating score of caregiver-reported challenges	0.492	0.492	0.181	
***Dependent variable (n** **=** **41)***	***Beta***	***Partial correlation***	***95%CI***	***P-value***
Blended feed (Dummy variable)^a^	2.44	0.484	−0.38 to −1.0	0.002^*^
Commercially available ready-made formulas (Dummy variable)^b^	–	–	–	–

^a^Predictors: (constant).

^b^Excluded variables.

All models are adjusted for child's age and gender.

^*^P-value is statistically significant at < 0.05 level.

## 4 Discussion

This pilot study provides a snapshot of caregiver-reported feeding and nutritional problems associated with long-term EN in children with disabilities. Overall, the findings indicated that the most frequently reported issues were constipation, weight loss, and GERD. Additionally, the indication for EN was a significant predictor of subsequent feeding and nutritional problems associated with long-term use. We also found a statistically significant association between dental problems and feeding difficulties in children with disabilities. Finally, the challenges experienced by caregivers were significantly related to the type of EN provided to the children.

Most children in the cohort were diagnosed with CP (34%), which aligns with current evidence indicating that CP is a common cause of disability among children in Saudi Arabia. Additionally, our data showed that constipation was the most frequently reported gastrointestinal issue associated with long-term EN. This finding is consistent with previous studies, which have estimated that up to 72% of children with CP experience chronic constipation, particularly when receiving prolonged enteral feeding (19, 20). Comparable data from a cross-sectional study examining the prevalence and characteristics of constipation among 152 pediatric patients with various disabilities indicated that constipation was the most commonly reported problem, regardless of medical interventions such as the use of laxatives (21). Children with CP are particularly vulnerable to chronic constipation due to several contributing factors, including medication side effects, overfeeding, limited mobility, improper positioning, and scoliosis, in addition to the adverse effects of neurological impairment (NI) on gut motility (22–24). Chronic constipation and diarrhea may also be triggered by gut microbiota dysbiosis associated with long-term enteral feeding (25). Evidence-based guidelines for managing chronic constipation in children with neurological impairment recommend a combination of adequate dietary fiber, the use of laxatives, sufficient fluid intake, and age-appropriate physical activity (23, 26–28). However, in children with severe gastrointestinal dysmotility who are unable to tolerate EN despite comprehensive interventions, parenteral nutrition may be considered as a last-resort option after exhausting all enteral feeding strategies and pharmacological treatments (24). It has been reported that approximately 80% to 90% of children with CP or neurodevelopmental disabilities are at high risk of developing malnutrition due to constipation and other gastrointestinal issues, such as uncoordinated swallowing and GERD (29).

Our data indicated that weight loss was also frequently reported by caregivers as a problem among children with disabilities receiving prolonged EN. This weight loss may, in part, be attributed to caregivers' limited knowledge in accurately estimating their children's nutritional requirements. In this context, our findings revealed that 25% of caregivers struggled to determine appropriate dietary needs for their children. Therefore, dietitians should work closely with families to develop individualized therapeutic nutrition plans and educate caregivers on the potential risks of infection or nutrient deficiencies that may arise from improper preparation or storage of tube-feeding formulas (24).

Consistent with the existing literature, our data indicated that GERD was frequently experienced by children in the study cohort following EN delivery. GERD and constipation are commonly observed in children with neurological impairment, regardless of the feeding method (23). This is largely due to dysfunction of the enteric nervous system, which regulates critical gastrointestinal functions such as motility, secretion, and blood flow (23, 26, 30). Children with neurological impairment often present with multiple coexisting risk factors that predispose them to severe and chronic GERD. These include reduced lower esophageal sphincter pressure, delayed gastric emptying, esophageal dysmotility, constipation, recurrent seizures, prolonged supine positioning, scoliosis, and adverse effects of medications (31). Failure to assess and manage GERD-related feeding problems at an early stage can accelerate the development of malnutrition, increase hospitalization rates, and further impair the quality of life of children with disabilities (23, 32, 33).

Interestingly, we found a statistically significant association between the indications for nutrition support and the type of feeding and nutritional problems subsequently reported among children with disabilities. For example, children who were prescribed EN due to inadequate oral intake were more likely to experience insertion site infections. This consistent with data from other studies where they reported issues with tube site infection, despite the overall safety of enteral feeding provision (31, 34). The increased risk of infection at the tube insertion site may be attributed to greater reliance on, and prolonged duration of, tube feeding to compensate for insufficient oral intake. Supporting our observation, a longitudinal study reported that tube-related complications such as dislodgment, peristomal leakage, and wound infections were common among patients receiving long-term home EN (35). In addition, inadequate food intake among children in our cohort may predispose them to subclinical micronutrient deficiencies, which can impair immune function and increase their susceptibility to infections, including at the tube insertion site (34). Supporting this observation, previous studies have reported that peristomal infections may result from weakened immune defenses. Recent recommendations also suggest the use of prophylactic antibiotics at the time of initial tube placement to enhance immunocompetence and reduce the risk of infection (36).

Among our cohort, tube-related complications, such as tube clogging, were less frequently reported by caregivers. This low frequency may indicate that caregivers had developed experience and familiarity with tube-feeding maintenance through prolonged practice. However, it may also point to a potential gap in their ability to recognize less apparent complications, such as partial tube occlusion, whereas more visible issues like insertion site infections were more readily identified and reported, as reflected in our findings. This area warrants further investigation in future studies to better understand caregiver awareness and recognition of tube-related complications.

Our data showed a significant association between dental problems and feeding problems associated with EN. The literature widely documents that inadequate oral care in long-term tube-fed patients with dysphagia can create a bacterial reservoir, thereby increasing the risk of aspiration (37). In fact, children with disabilities have more complex oral healthcare needs compared to their healthy peers (38). Substantial evidence suggests that such unfavorable alterations in salivary composition may increase the risk of feeding-related complications, particularly aspiration pneumonia (12). As long-term EN can disrupt the oral ecosystem by disturbing the balance of indigenous oral microbiota, allowing pathogenic bacteria to thrive an imbalance associated with an elevated risk of aspiration (37). For example, Huang et al. (37) reported that tube-fed patients with dry mouth had a 4.23 times higher risk of aspiration than those without dry mouth. In turns, Increased reliance on enteral feeding reduces regular oral stimulation both mechanical (e.g., chewing) and chemical (e.g., tasting) which in turn leads to diminished saliva production and lower oral pH levels. These changes promote tooth decay due to food debris and plaque accumulation (12). It has also been reported that irreversible loss of dental hard tissue and dissolution of mineralized tooth structure occur in chronic GERD patients, resulting from frequent contact with acids introduced into the oral cavity from the stomach (39, 40). Given the bidirectional relationship between oral pathological manifestations and the prediction of GERD or EN complications, careful oral assessment should be an integral part of managing children receiving long-term EN.

Finally, caregivers were asked two questions to assess the level of challenges and difficulties they faced in managing EN for their children. The results indicated that the type of EN provided was a significant predictor of the level of difficulty reported. Specifically, home-blended food was statistically associated with higher challenge scores on the Likert scale compared to ready-made formulas. This finding aligns with previous research suggesting that ready-made enteral formulas are generally more convenient and easier for caregivers to handle than home-blended alternatives (11). Although home-blended food is often perceived as a more natural and cost-effective option for EN, it typically provides lower caloric and micronutrient content compared to ready-to-use formulas and carries a higher risk of bacterial contamination due to home preparation (11, 41). Additionally, caregivers may face challenges in preparing nutritionally balanced blenderized meals that meet all of their child's energy and nutrient requirements. Inadequate caloric intake can impair growth and limit weight gain in children with disabilities (42). Despite the associated challenges, existing literature has suggested that home-blended diets may help alleviate various gastrointestinal symptoms such as GERD, constipation, abdominal discomfort, nausea, and vomiting (43, 44). Some studies recommend a combined approach using both blenderized feeds and commercial formulas to help reduce tube-feeding-related symptoms while supporting oral intake and promoting growth (24).

Given the central role caregivers play in managing EN at home, their ability to recognize, respond to, and communicate complications is crucial for optimizing clinical outcomes. Importantly, this study highlights the need to develop targeted interventional strategies that empower caregivers to detect and address EN-related issues early. These may include caregiver-centered educational programs focusing on the signs and symptoms of common complications such as constipation, regurgitation, and inadequate weight gain, as well as guidance on proper feeding techniques and hygiene practices. In addition, individualized consultation to help them prepare home-blended meals that are calorically and nutrient-dense, safe for tube feeding, and free from potential sources of microbiological contamination. The integration of structured monitoring tools or caregiver checklists may enhance communication with healthcare providers and reduce the risk of unrecognized complications. Although the present study did not evaluate formal monitoring protocols, its caregiver-reported data serve as an important foundation for designing caregiver screening instruments and follow-up systems. Embedding such tools within routine care particularly at discharge and during outpatient follow-up may not only improve complication tracking but also alleviate caregiver burden and anxiety. Future research should explore the development and validation of home-based screening protocols, which could include digital reporting platforms, visual symptom trackers, or nurse-led telehealth check-ins. Ultimately, promoting caregiver education and involvement in EN monitoring can contribute to safer, more responsive, and family-centered nutrition support services.

To our knowledge, this is the first study in the region to explore, from the caregivers' perspective, the complications associated with long-term EN among disabled children and the challenges faced by caregivers in handing EN. While the present study offers valuable insights into feeding complications among children receiving long-term enteral nutrition, it is important to acknowledge the limitations inherent in the scope of the data collected. The use of a validated tool guided the focus toward caregiver-reported complications and basic information regarding feed types, without capturing more granular clinical variables such as the type of feeding tube (e.g., nasogastric, gastrostomy), feeding schedules (e.g., bolus vs. continuous), duration of EN therapy, or concurrent comorbidity management strategies. These factors are known to potentially influence the type and severity of complications experienced. Future studies should aim to expand the set of variables collected to enable subgroup analyses and facilitate a more comprehensive characterization of EN practices. Doing so would allow for deeper understanding of how specific clinical interventions relate to patient outcomes and could help optimize protocols for different patient populations. The river sampling technique used to recruit sample may limit the study generalizability. Additionally, the self-reported nature of the data may introduce reporting bias, nevertheless, the questionnaire used was previously developed and validated in earlier study, enhancing the reliability of the findings. Another limitation of this study is the relatively small sample size. However, as this was designed as a pilot study, the primary aim was to generate preliminary data, explore feasibility, and identify potential trends rather than to provide definitive conclusions. The findings should therefore be interpreted with caution and considered as a basis for informing larger, adequately powered future studies. Despite these limitations, the results offer valuable insights into the challenges of prolonged EN in pediatric populations, particularly within Saudi Arabia.

## 5 Conclusion

The current study highlighted many feeding and nutritional problems associated with prolonged EN among children with disabilities in Saudi Arabia, such as constipation, vomiting, and infection on tube feeding insertion sit. In addition, the results drew attention to the association of prolonged EN, oral health, and the adequacy of oral intake among disabled children. We also shed light on the challenges experienced by the caregivers of children receiving long-term EN. Optimizing tube feeding facilitates optimizing nutritional status and growth, preventing malnutrition, and improving the overall health-related life quality of disabled children and their caregivers and families. Further research with larger, more diverse samples is recommended to establish evidence-based guidelines for managing EN-related feeding and nutritional complications in this vulnerable population.

## Data Availability

The raw data supporting the conclusions of this article will be made available by the authors, without undue reservation.
